# Formation and Evolution of Micro/Nano Periodic Ripples on 2205 Stainless Steel Machined by Femtosecond Laser

**DOI:** 10.3390/mi14020428

**Published:** 2023-02-11

**Authors:** Xiaofeng Xu, Laifei Cheng, Xiaojiao Zhao, Jing Wang, Ke Tong, Hua Lv

**Affiliations:** 1 Science and Technology on Thermostructural Composite Materials Laboratory, School of Materials Science and Engineering, Northwestern Polytechnical University, Xi’an 710072, China; 2 CNPC Tubular Goods Research Institute, Xi’an 710077, China; 3 School of Electronic Engineering, Xi’an Shiyou University, Xi’an 710065, China

**Keywords:** femtosecond laser, micro/nano ripples, formation, evolution, stainless steel, pea pod-like structure

## Abstract

The preparation of micro/nano periodic surface structures using femtosecond laser machining technology has been the academic frontier and hotspot in recent years. The formation and evolution of micro/nano periodic ripples were investigated on 2205 stainless steel machined by femtosecond laser. Using single spot irradiation with fixed laser fluences and various pulse numbers, typical ripples, including nano HSFLs (‖), nano LSFLs (⊥), nano HSFLs (⊥) and micro grooves (‖), were generated one after another in one test. The morphologies of the ripples were analyzed, and the underlying mechanisms were discussed. It was found that the nano holes/pits presented at all stages could have played a key role in the formation and evolution of micro/nano periodic ripples. A new kind of microstructure, named the pea pod-like structure here, was discovered, and it was suggested that the formation and evolution of the micro/nano periodic ripples could be well explained by the pea pod-like structure model.

## 1. Introduction

Femtosecond laser machining has attracted widespread attention because micro/nano periodic surface structures can be produced in a convenient way through this kind of advanced technology [1,2,3,4,5,6,7,8,9,10]. Similar to the natural hierarchical structures found in our world, the man-made micro/nano periodic surface structures have been proven to have a significant influence on material surface properties, such as adjusting wetting performance [11,12,13], enhancing light absorption [14,15,16] and strengthening tribological and corrosion properties [17,18,19,20], thus demonstrating their bright application prospects in high-end manufacturing fields.

By controlling the experimental environment and processing parameters, various kinds of micro/nano periodic surface structures have been prepared [21,22,23]. Among them, typical ripple structures are a subject of greatest interest. According to the spatial period and direction, ripple structures usually include three categories, namely, nanoscale low-spatial-frequency LIPSS (LSFL) with spatial periods close to the irradiation wavelength and orientation perpendicular to the laser beam polarization, nanoscale high-spatial-frequency LIPSS (HSFL) with spatial periods less than half the wavelength and microscale groove structures with orientations parallel to the laser beam polarization. To understand the mechanisms of the formation of these ripples, several explanation models have been proposed, such as interference [24,25], surface plasma [26,27], second harmonic generation [28,29], self-organization [1,30] and Coulomb explosion [31]. However, the mechanism remains an open question, as there are still many interesting observations that cannot be clearly and fully explained. The formation process of these ripple structures is a complex non-linear, non-equilibrium process involving many optical, thermodynamic and mechanical principles. There is still much to be understood about the formation and evolution of these micro/nano structures. For example, the existing studies have verified the transformation of nano HSFLs (‖) to nano LSFLs (⊥) [5], nano LSFLs (⊥) to micro grooves (‖) [2] and nano LSFLs (⊥) to nano HSFLs (⊥) [28,32], respectively. Based on these results, it is interesting to infer that the transition of nano HSFLs (‖) to nano LSFLs (⊥) and then to a micro ripple/groove (‖) may be a continuous transition process. Therefore, more experiments and theoretical studies should be conducted to further comprehend the formation and mechanism of the ripples.

The morphology and the spatial periods of the ripples vary with many factors, including the physical and chemical properties of the material itself, the laser physical parameters and the processing parameters [23]. In this work, we investigated the effects of pulse numbers on surface morphology changes in stainless steel. Typical ripple structures were obtained, the morphologies of the craters and ripples were investigated, the evolution of the ripples was analyzed and the underlying mechanisms were discussed.

## 2. Experimental Process

**Figure 1:**
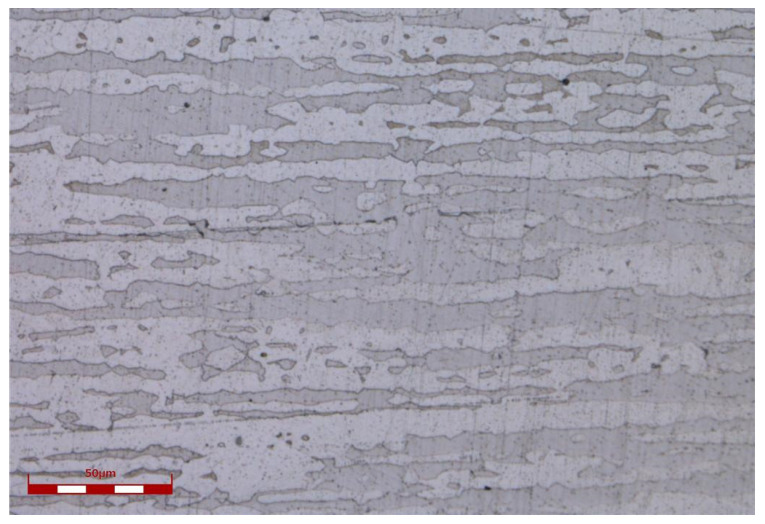
The duplex microstructure (α-ferrite and austenite) of the test material.

2205 duplex stainless steel specimens with dimensions of 15 mm × 15 mm × 2 mm were used as targets, which were mirror finished by mechanical polishing with 1500# sandpaper, and then fine polishing with 2.5 μm diamond powder. The chemical composition of the material is shown in Table 1 and the microstructure is shown in Figure 1.

**Figure 2:**
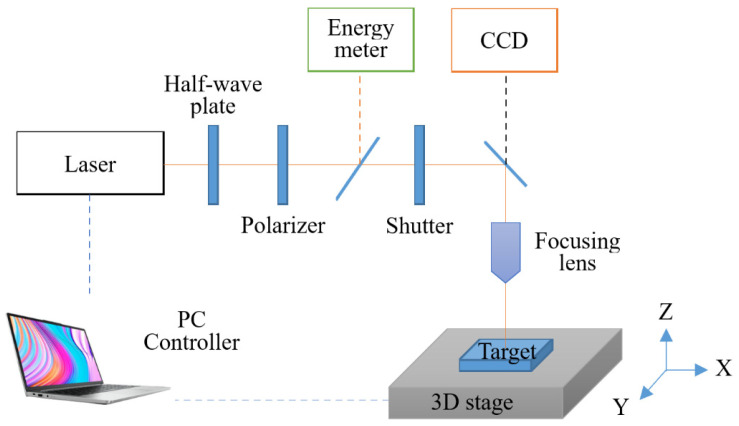
Schematics of the experimental femtosecond laser system for machining.

As shown in Figure 2, a commercial femtosecond laser system (Pharos, Light Conversion, Lithuania) was used to generate linearly polarized laser pulses, with a central wavelength of 1030 nm and a pulse duration of 300 fs. The Gaussian-profiled laser was focused by a 100 mm-focal-length lens directly onto the surfaces of the samples with a radius of *w*_0_ = 19.5 μm (as measured at 1/e^2^ of the maximum intensity). The laser fluence of the incident light was adjusted through a combination of a half-wave plate and a linear polarizer, and an energy detector was used to measure the laser-pulse energy. The number of pulses was controlled by a commercial electronic shutter. The stainless steel surface was irradiated in single spot irradiation mode at a fixed laser fluence (0.4 J/cm^2^) and various pulse numbers (20, 40, 80, 160, 320, 640 and 1280 pulses). A repetition rate of 1 kHz was employed in this research. The specimen was fixed to a computer-controlled high-precision 3D stage (Aerotech ANT 130), allowing the precise positioning of the specimen between the irradiation sequences. All irradiations were performed under normal incidence in an atmospheric environment at room temperature.

After laser machining, the morphologies of the craters were characterized using a 3D laser confocal microscope (OLS 4100, Olympus, Japan), the formation and evolution of the micro/nano structures were examined using a scanning electron microscope (SEM, Supra 55, Zeiss, Germany) and the spatial periods of the ripples were processed with the assistance of the image processing software Fiji.

## 3. Results and Discussion

### 3.1. LIPSS and Crater Morphology

**Figure 3:**
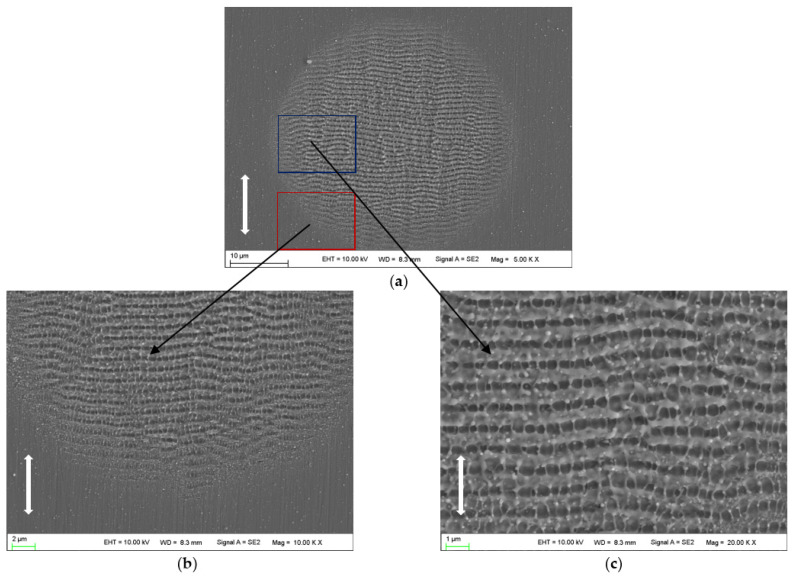
SEM images of nanostructures on the surface irradiated by 20 laser pulses: (**a**) overview of the whole ablated area; (**b**) peripheral region with higher magnification; (**c**) central region with higher magnification. The vertical arrows indicate the polarization orientation of the incident laser beam.

Figure 3 shows the morphologies on the 2205 stainless steel surface irradiated by 20 laser pulses at a laser fluence of 0.4 J/cm^2^. In the whole ablation region, it is clearly seen that the surface is covered by homogenous LSFLs (⊥), which have an orientation perpendicular to the laser beam polarization and spatial periods of 890~940 nm. In addition to LSFLs (⊥), ultrafine HSFLs (‖) are observed, which have an orientation parallel to the polarization direction of the laser beam and spatial periods of 380~470 nm. This kind of structure is too small to be observed at low magnification.

**Figure 4:**
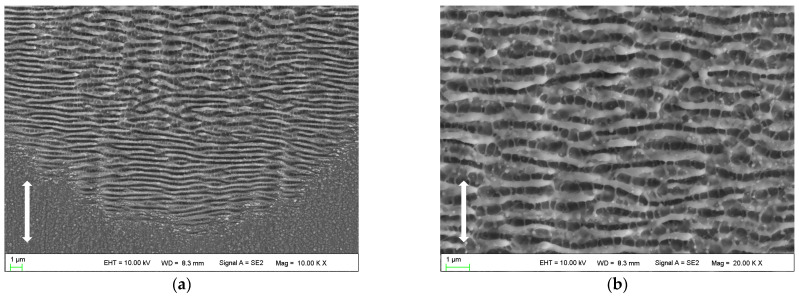
SEM images of nanostructures on the surface irradiated by 80 laser pulses: (**a**) peripheral region; (**b**) central region. The vertical arrows indicate the polarization orientation of the incident laser beam.

With the pulse number reaching 80, distinct and orderly arranged HSFLs (⊥) are formed in the peripheral region of the laser irradiated spot as shown in Figure 4a. The orientation of this kind of HSFL (⊥) is perpendicular to the polarization direction of the laser beam, with a spatial period of approximately 430 nm. The ripple structures in the center of the laser irradiated region are still LSFLs (⊥), which become discontinuous and irregular, and the spatial period is approximately 645 nm as shown in Figure 4b. Ultrafine HSFLs (‖) can also be observed both in the central and peripheral regions, with spatial periods of 280~400 nm.

**Figure 5:**
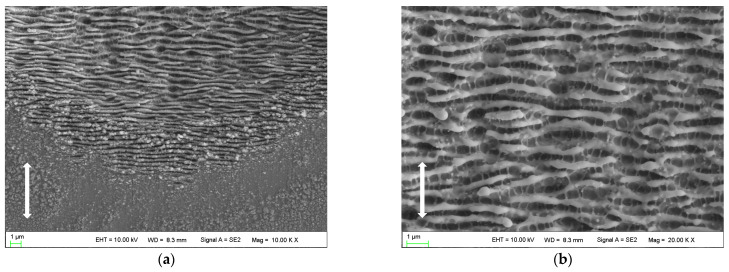
SEM images of micro/nanostructures on the surface irradiated by 320 laser pulses: (**a**) peripheral region; (**b**) central region. The vertical arrows indicate the polarization orientation of the incident laser beam.

With the number of pulses reaching 320, micro grooves (‖) are clearly observed in the center of the ablated region with an orientation parallel to the polarization direction of the laser beam and a spatial period of approximately 3.4 μm, as shown in Figure 5b. Due to the presence of micro grooves (‖), the LSFLs (⊥) in the center are truncated into short segments, but the spatial period along the polarization direction of the laser beam remains similar to that in Figure 4. Compared with Figure 4, the HSFLs (⊥) in the peripheral region and ultrafine HSFLs (‖) observed in the center do not have significant changes, with spatial periods of approximately 400 nm and 350 nm, respectively. However, much more debris and dust cover the HSFLs (⊥) in the peripheral region and are observed around the ablated area.

### 3.2. Evolution of the Periodic Structures

According to the findings of this work as shown in Figure 3, Figure 4 and Figure 5, it was found that LSFLs (⊥) and ultrafine HSFLs (‖) are formed first; then, with the increase in laser pulses, HSFLs (⊥) and micro grooves (‖) are prepared one after another. All those desired typical micro/nano ripples have been formed in one test by changing the laser pulses.

The formation of LSFLs (⊥) is generally explained by the interference mechanism [24,33]. The ripples can be generated by locally modulated ablation resulting from interference between the incident laser beam and surface plasmon polaritons (SPPs) or scattered waves from the surface undulations. According to this model, the ripples’ spatial period is predicted to be approximately equal to the laser wavelength at a normal incidence. Here, the spatial periods of LSFLs (⊥) appearing in Figure 3 are very close to the laser wavelength, and it is likely that the interference mechanism is responsible for the formation of such ripples at this stage.

**Figure 6:**
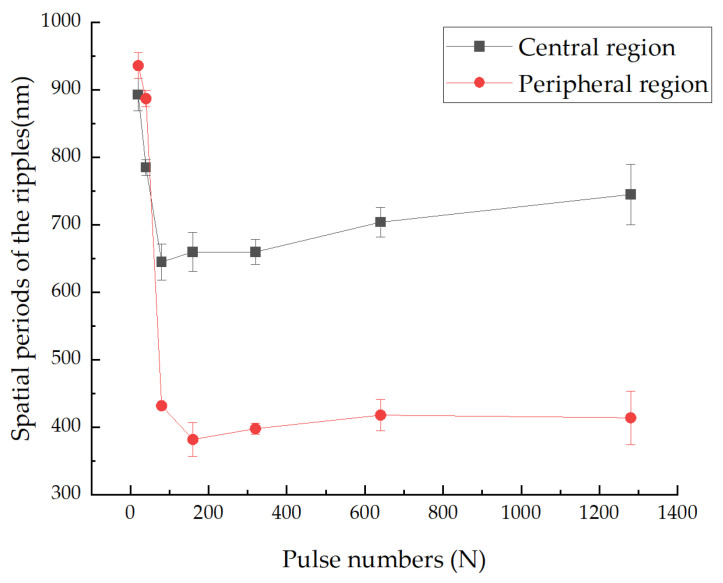
Spatial periods of the ripples as a function of the pulse numbers.

**Figure 7:**
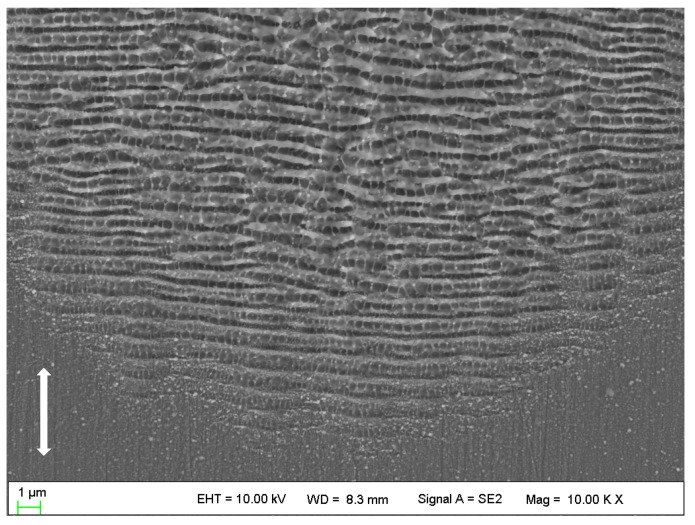
SEM images of nanostructures irradiated by 40 laser pulses in the peripheral region. The vertical arrows indicate the polarization orientation of the incident laser beam.

Interestingly, as the number of pulses increases, the spatial periods of the ripples in the peripheral region decrease rapidly. After reaching approximately 400 nm, however, it no longer changes, forming new orderly arranged HSFLs (⊥), as shown in Figure 4a, Figure 5a and Figure 6. The formation of HSFLs (⊥) could be attributed to the split of LSFLs (⊥), and this phenomenon can be clearly observed in Figure 7. The same results were obtained by Liu B. et al. [32] and Hou S. et al. [28]. This may be caused by the non-linear effect of second harmonic generation (SHG) [26,29]. The plasma layer induced by the ultrashort laser beam cannot substantially expand in a short time, and then the interaction between this thin plasma layer and subsequent laser pulses leads to SHG. Hou S. et al. [28] made a theoretical calculation to study the ultrafast dynamics of the formation of ripples and proved the variation in electric field intensity which may result in the division of ripples. Although the spatial period decreases, the ripples in the center region of the ablated spot remain as LSFLs (⊥). This may be because of the near Gaussian characteristics of the laser beam, making the ripples much stronger and wider.

When the number of pulses surpasses 320, micro grooves (‖) are clearly observed in the center of the ablated region as shown in Figure 5b. This kind of microstructure is characterized by rows of nano scale holes arranged in an orderly manner along the polarization direction of the laser beam with an average spacing between the rows of approximately 3~4 μm. The formation of the arranged holes may involve the Coulomb explosion during local spatially modulated ablation [31,32].

HSFLs (‖) emerge from the very beginning and are observed at all stages. However, the formation mechanism of HSFLs (‖) is under a lot of debate [3,32]. When examining carefully, it is inspiring to find that the ripples are also composed of a small set of regularly arranged holes/pits. Hence, it is inferred that the holes/pits must have played a key role in the formation and evolution of the micro/nano periodic ripples. 

### 3.3. Supplementary Test and New Findings

The single pulse ablation threshold *F*_th_(1) calculated using the *D*^2^-ln*F* method and accumulation model [34] for 2205 stainless steel was approximately 0.17 J/cm^2^. Ablation occurs when the irradiation laser fluence is higher than the threshold value. By using 0.2 J/cm^2^, slightly higher than the ablation threshold, the preliminary structure is observed with few laser pulses and some interesting clues are obtained.

**Figure 8:**
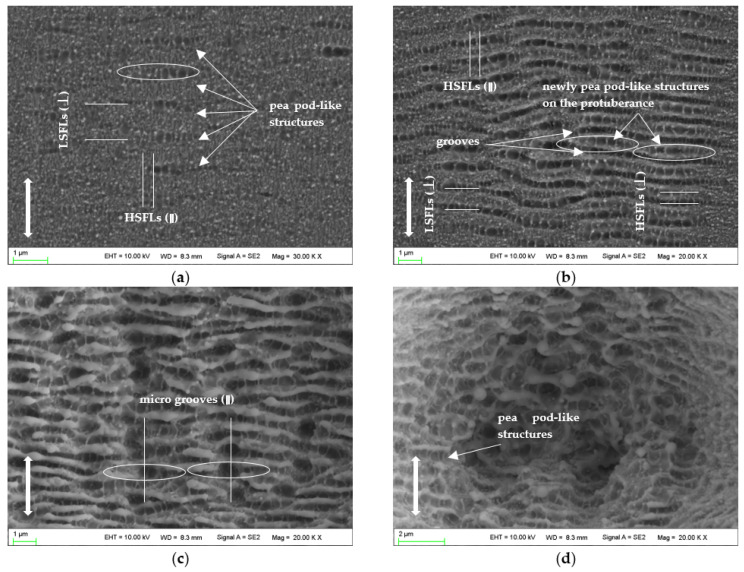
SEM images of nanostructures irradiated by 0.2 J/cm^2^: (**a**) 20 pulses; (**b**) 40 pulses; (**c**) 640 pulses; (**d**) 1280 pulses. The vertical arrows indicate the polarization orientation of the incident laser beam.

Figure 8 shows the morphologies irradiated by dozens of laser pulses at a laser fluence of 0.2 J/cm^2^. When the pulse number is 20, several pea pod-like structures emerge, consisting of some regularly arranged holes/pits with a diameter of 300~400 nm, as shown in Figure 8a. At this stage, the LIPSSs are not maturely developed, but parts of the structure characteristics of HSFLs (‖) and LSFLs (⊥) are observed.

When the pulse number reaches 40, as shown in Figure 8b, the early-formed pea pod-like structures join to form the grooves and continuous LSFLs (⊥); and the newly formed pea pod-like structures are generated on the protuberance between the two grooves to form HSFLs (⊥). Each hole/pit of the pea pod-like structures is a part of HSFL (‖); and when the ablated area is covered by LIPSSs (⊥), the morphology of HSFL (‖) becomes clear. When examining carefully, we may find the discontinuous characteristics of HSFL (‖).

Meanwhile, it is noted that the lengths of the pea pod-like structures are approximately 3~4 μm, and the hole/pit in the middle is the largest in the pea pod-like structure. This indicates that the middle of the pea pod-like structure obtains the highest energy, and as the number of pulses increases, this place burns the most severely, and eventually forms deeper holes, as shown in Figure 8c. This corresponds to the formation process of micro grooves (‖). The pea pod-like structure even can be observed when the material is irradiated at 1280 pulses, as shown in Figure 8d.

**Figure 9:**
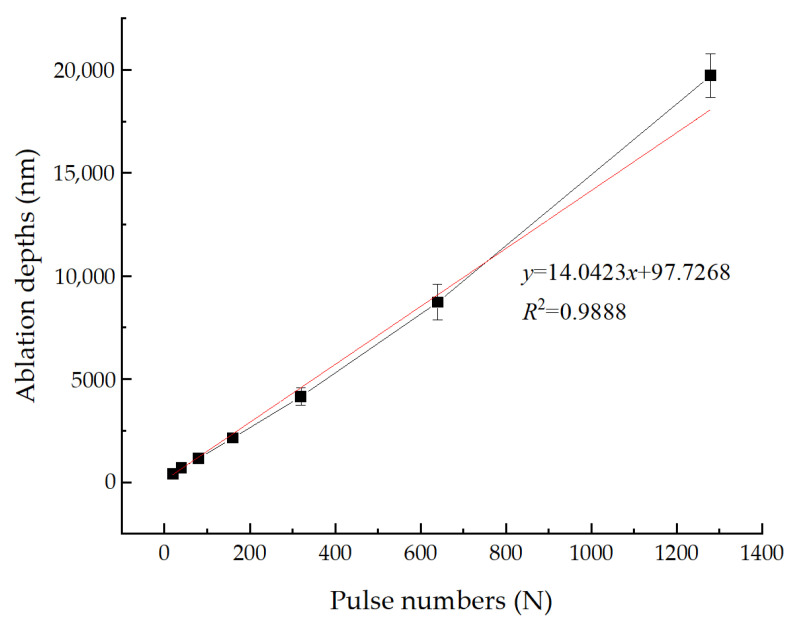
Ablation depths as a function of the pulse numbers at 0.4 J/cm^2^.

**Figure 10:**
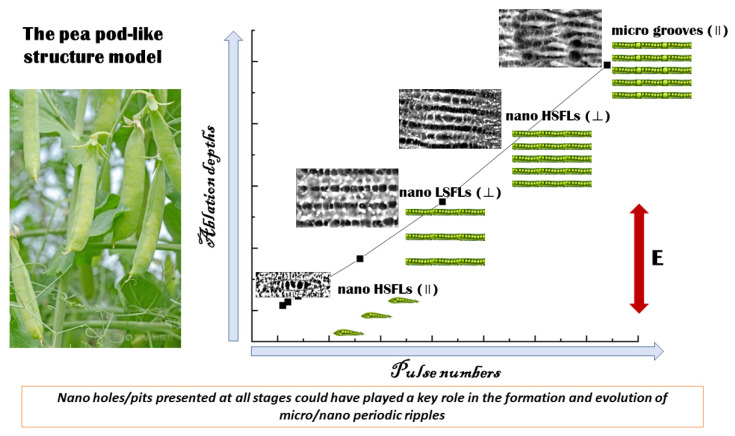
This is a figure. Schemes follow the same formatting.

In addition, the ablation depths obtained by measuring the maximum depths of the craters are investigated. Figure 9 shows the ablation depths of 2205 stainless steel varying with the pulse numbers in air at 0.4 J/cm^2^. A near-linear relationship is found between the ablation depths and the irradiation pulse numbers, suggesting that the main ablation mechanism for the formation and evolution of those periodic ripples may be the same. The pea pod-like structure, a relatively stable structure and found in many stages, might be an intermediate transition structure, and could be used to deduce the evolution of the ripples based on the findings of this paper, as shown in Figure 10.

On the one hand, the periodic characteristic of the ripples (⊥) could be related to the wave characteristic and interference phenomena, which are widely accepted. Specifically, for nano LSFLs (⊥), the formation could be attributed to the interference mechanism, while the formation of nano HSFLs (⊥) could be attributed to the effect of second harmonic generation (SHG). On the other hand, when examining carefully, we may find the nano holes/pits have approximately spherical features and the walls of the nano dots/holes are not ablated, which indicate the surface is ablated by discrete energy.

The formation and evolution of the micro/nano periodic structures are complex. So far, there is no unified mechanism which can explain the characteristics of all periodic structures. In this study, the evolution of micro/nano periodic structures is explained through the pea pod-like structure, which provides a new idea and approach for the evolution of micro/nano periodic structures. Of course, a lot of in-depth numerical simulation and theoretical analysis are needed in the future to provide theoretical support for the formation and evolution of micro/nano periodic structures.

## 4. Conclusions

The formation of micro/nano periodic structures on 2205 stainless steel machined by a femtosecond laser has been experimentally studied. Those typical ripples, including nano HSFLs (‖), nano LSFLs (⊥), nano HSFLs (⊥) and micro grooves (‖), were generated by using the single spot irradiation mode at fixed laser fluences (0.2, 0.4 J/cm^2^) and various pulse numbers (20, 40, 80, 160, 320, 640 and 1280 pulses). The morphologies of those ripples have been observed, and the evolution mechanisms have been analyzed. For nano LSFLs (⊥), the spatial periods were approximately 890~940 nm at 20 pulse numbers, and the formation could be attributed to the interference mechanism. Nano HSFLs (⊥) formed when the pulse numbers exceeded 80 and the spatial periods were approximately 400~430 nm. Under the effect of second harmonic generation, the split of nano LSFLs (⊥) played a key role in the formation of nano HSFLs (⊥). For micro grooves (‖), the spatial periods were approximately 3~4 μm, and the Coulomb explosion could be involved in the formation of the arranged holes at the sides of the microstructure. Nano HSFLs (‖) emerged at the very beginning and were observed at all stages. A pea pod-like structure was found in the supplementary test, and it was suggested that the formation and evolution of the micro/nano periodic ripples could be well explained by the pea pod-like structure proposed in this work.

## Figures and Tables

**Table 1:** Main element chemical composition of the carbon steel, Wt.%.

C	Si	Mn	Cr	Mo	Ni	Nb	V	Ti	Cu	Al
0.02	0.53	1.49	22.10	2.93	5.50	0.009	0.110	0.008	0.15	0.09

## Data Availability

No new data were created or analyzed in this study. Data sharing is not applicable to this article.
